# Supplementary material for: Longitudinal electrophysiological changes after mesenchymal stem cell transplantation in a spinal cord injury rat model
Source: PLoS One. 2022 Aug 5;17(8):e0272526. doi: 10.1371/journal.pone.0272526 (PMC9355172; doi:10.1371/journal.pone.0272526)
Supplement: S1 Table — (DOCX) [file pone.0272526.s001.docx]

**S1 Table. Surface marker expression in MSCs from various tissues.**

| **MSC positive markers (%)** | **rbMSCs** | **rcMSCs** |
| --- | --- | --- |
| **CD29** | 98.05 ± 0.55 | 99.80 ± 0.20 |
| **CD44** | 99.90 ± 0.10 | 99.65 ± 0.25 |
| **CD90** | 95.05 ± 1.85 | 93.80 ± 1.60 |
| **MSC negative markers (%)** |  |  |
| **CD34** | 1.16 ± 0.33 | 0.73 ± 0.06 |
| **CD45** | 0.35 ± 0.02 | 0.29 ± 0.04 |

Data represent the mean ± SD of independent experiments (n = 2).

Abbreviations: rbMSCs: bone marrow-derived MSCs; rcMSCs: cranial bone-derived MSCs; SD: standard deviation.
