# Supplementary figures and images for: Longitudinal electrophysiological changes after mesenchymal stem cell transplantation in a spinal cord injury rat model
Source: PLoS One. 2022 Aug 5;17(8):e0272526. doi: 10.1371/journal.pone.0272526 (PMC9355172; doi:10.1371/journal.pone.0272526)

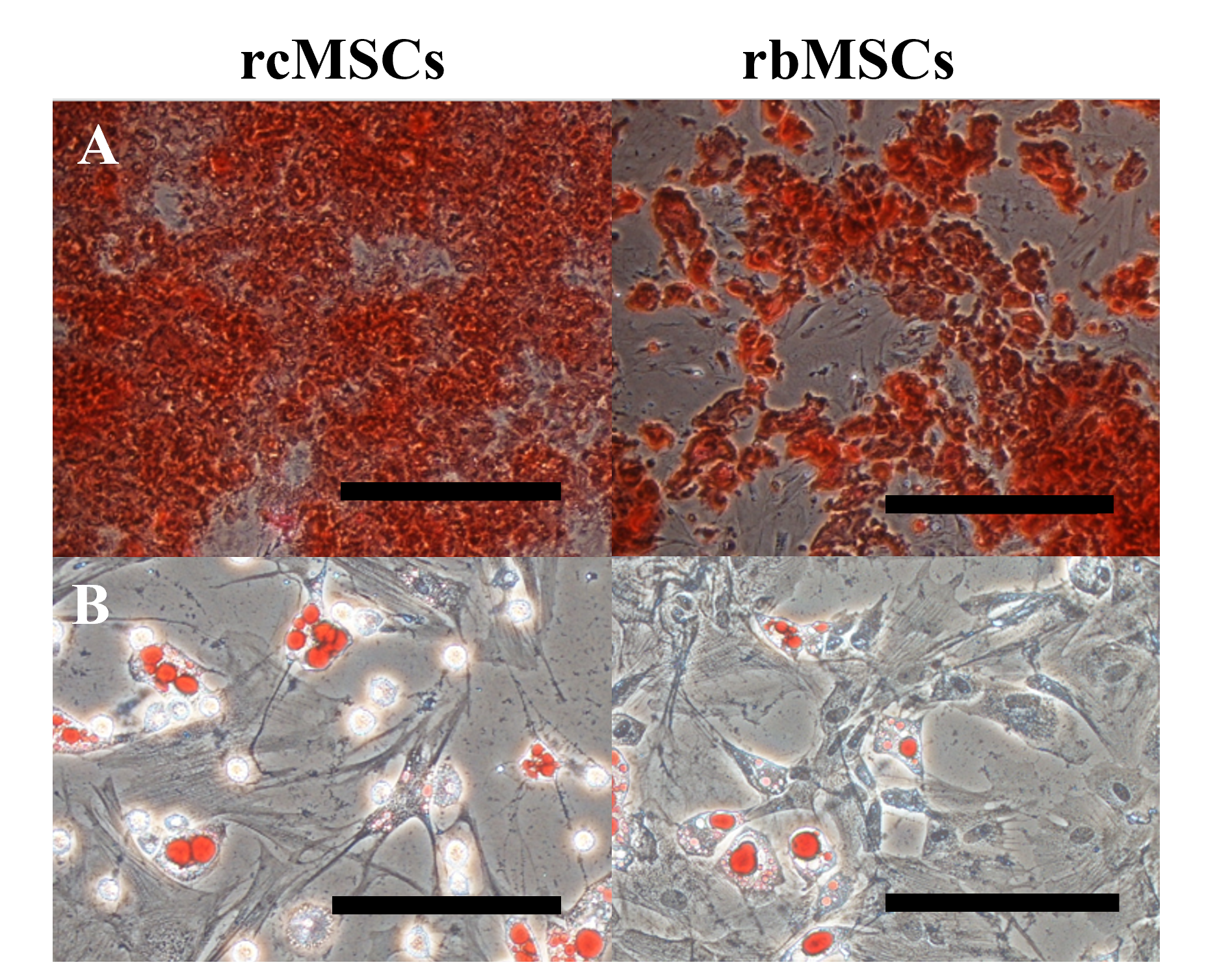

Supplement: S1 Fig — (A) Alizarin red S staining following the osteogenic differentiation of rbMSCs and rcMSCs. (B) Oil red O staining following the adipogenic differentiation of rbMSCs and rcMSCs. Scale bars, 50 μm. (DOCX) [file pone.0272526.s002.docx]
